# Cyclophilin A Promotes Osteoblast Differentiation by Regulating Runx2

**DOI:** 10.3390/ijms23169244

**Published:** 2022-08-17

**Authors:** Meiyu Piao, Sung Ho Lee, Myeong Ji Kim, Joong-Kook Choi, Chang-Yeol Yeo, Kwang Youl Lee

**Affiliations:** 1 College of Pharmacy, Chonnam National University, Gwangju 61186, Korea; 2 College of Pharmacy, Research Institute of Pharmaceutical Sciences, Chonnam National University, Gwangju 61186, Korea; 3 Division of Biochemistry, College of Medicine, Chungbuk National University, Cheong-Ju 28644, Korea; 4 Department of Life Science, Research Center for Cellular Homeostasis, Ewha Woman’s University, Seoul 03760, Korea

**Keywords:** cyclophilin A, osteoblast, Runx2, Akt signaling

## Abstract

Cyclophilin A (CypA) is a ubiquitously expressed and highly conserved protein with peptidyl-prolyl *cis-trans* isomerase activity that is involved in various biological activities by regulating protein folding and trafficking. Although CypA has been reported to positively regulate osteoblast differentiation, the mechanistic details remain largely unknown. In this study, we aimed to elucidate the mechanism of CypA-mediated regulation of osteoblast differentiation. Overexpression of CypA promoted osteoblast differentiation in bone morphogenic protein 4 (BMP4)-treated C2C12 cells, while knockdown of CypA inhibited osteoblast differentiation in BMP4-treated C2C12. CypA and Runx2 were shown to interact based on immunoprecipitation experiments and CypA increased Runx2 transcriptional activity in a dose-dependent manner. Our results indicate that this may be because CypA can increase the DNA binding affinity of Runx2 to Runx2 binding sites such as osteoblast-specific *cis*-acting element 2. Furthermore, to identify factors upstream of CypA in the regulation of osteoblast differentiation, various kinase inhibitors known to affect osteoblast differentiation were applied during osteogenesis. Akt inhibition resulted in the most significant suppression of osteogenesis in BMP4-induced C2C12 cells overexpressing CypA. Taken together, our results show that CypA positively regulates osteoblast differentiation by increasing the DNA binding affinity of Runx2, and Akt signaling is upstream of CypA.

## 1. Introduction

Cyclophilin A (CypA) is the prototypical member of the peptidyl-prolyl isomerase (PPIase) protein family, and it catalyzes the isomerization of peptidyl-prolyl bonds (Xaa-Pro bonds) and conformational changes during *cis-trans* protein folding. CypA was initially identified as a major intracellular target of the immunosuppressant drug cyclosporine (CsA), which forms a complex with CypA, thereby affecting immune responses [1,2]. Subsequent studies have shown that CypA is more often involved in the regulation of biological processes as molecular “switches” and in the regulation of the cell cycle, signal transduction, and gene expression [3]. Previous studies have shown that CypA deficiency in mice exerts a dual role on osteoblasts and osteoclasts, and this was shown to be due to CypA regulation of Smad1/5/8 phosphorylation and the recruitment of Smad4 and Runx2 [4]. However, the underlying mechanisms of CypA involvement in bone metabolism, particularly osteogenesis, remain unknown.

Runx2, also known as core-binding factor α1 (Cbfa1), is a Runt domain transcriptional activator. The highly conserved Runt homologous DNA binding domain of Runx2 can bind to the identical DNA sequence (5′-PyGPyGGTPy-3′) [5]. Runx2/Cbfa1 is widely expressed in osteoblasts and is essential for osteoblast differentiation. Runx2 regulates osteoblast differentiation by binding to osteoblast-specific *cis*-acting element 2 (OSE2), which is present in the *osteopontin (OPN)*, *collagen*, *bone sialoprotein (BSP)*, and *osteocalcin (OC)* promoters [6,7]. The expression and activation of the osteoblast-specific transcription factor Runx2 are regulated by several signaling pathways, including bone morphogenic protein (BMP), Wnt, and Hedgehog signaling [8]. Post-translational modifications such as phosphorylation, ubiquitination, and acetylation may affect the interaction between Runx2 and other nuclear factors [9,10].

Our study focused on the molecular mechanisms by which CypA regulates osteoblast differentiation. In detail, we discovered molecular targets for CypA and its upstream signaling involved in CypA-regulated osteoblast differentiation.

## 2. Results

### 2.1. CypA Enhances BMP4-Induced Osteoblast Differentiation In Vitro

**Figure 1:**
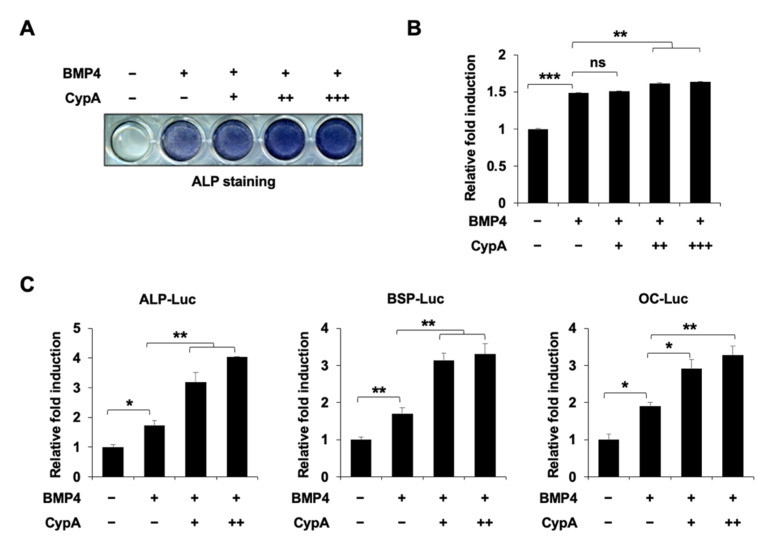
CypA enhances BMP4-induced osteoblast differentiation. (**A**,**B**) CypA significantly enhanced BMP4-induced ALP activity in a dose-dependent manner. C2C12 cells were cultured in DMEM (containing 10% FBS) and transfected with an empty vector or increasing amounts of *CypA* expressing plasmid using PEI transfection reagent. After 24 h, cells were removed from 10% FBS-containing DMEM and were maintained in a differentiation medium (containing 2% FBS), treated with or without BMP4 for 72 h. The ALP staining was performed to detect ALP activity. (**C**) CypA dose-dependently enhanced the luciferase activity of *ALP*, *BSP*, and *OC*. C2C12 cells were transfected with *ALP*, *BSP*, *OC*-Luc reporter genes (0.3 μg), β-galactosidase (0.05 μg), and an empty vector or increasing amounts of CypA. The cells were then stimulated with or without BMP4 for 36 h and luciferase activities were measured. Data are expressed as mean ± SEM of at least three experiments. *** *p* < 0.001, ** *p* < 0.01, and * *p* < 0.05 compared to the control group.

In previous studies [4], it was shown that *CypA*-deficient mice exhibited a reduction in both bone mineral density and osteoblast numbers. First, we analyzed the expression of CypA during the differentiation progression of C2C12 cells. We observed a ubiquitous expression of CypA during differentiation (Supplementary Figure S1). To investigate the role of CypA in osteoblast differentiation in vitro, we overexpressed *CypA* in BMP4-induced C2C12 cells. In the ALP staining results, the overexpression of *CypA* significantly increased BMP4-induced ALP activity (Figure 1A,B). Next, we measured the effect of CypA on *ALP*, *BSP*, and *OC* promoter activity by luciferase assay. CypA dose-dependently stimulated BMP4-induced promoter activity (Figure 1C). These results indicate that CypA consistently enhances the BMP4-induced differentiation of C2C12 cells.

### 2.2. Knockdown of CypA Reduces Osteoblast Differentiation

**Figure 2:**
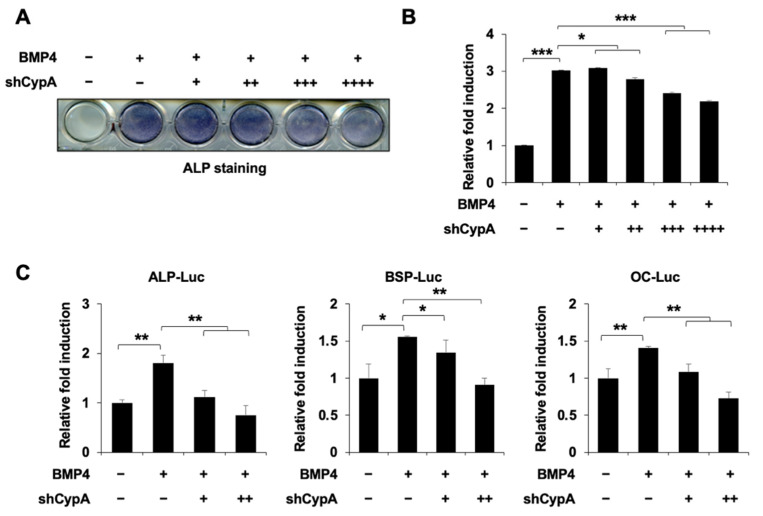
Knockdown of CypA reduces BMP4-induced osteoblast differentiation. (**A**,**B**) Knockdown of *CypA* significantly suppressed BMP4-induced ALP activity in a dose-dependent manner. C2C12 cells were cultured in DMEM (containing 10% FBS) and transfected with an empty vector (pSuper vector) or increasing amounts of shCypA plasmid using PEI transfection reagent. After 24 h, cells were removed from 10% FBS-containing DMEM and were maintained in a differentiation medium (containing 2% FBS), treated with or without BMP4 for 72 h. The ALP staining was performed to detect ALP activity. (**C**) Knockdown of *CypA* dose-dependently reduced the luciferase activity of *ALP*, *BSP*, and *OC*. C2C12 cells were transfected with *ALP*, *BSP*, *OC*-Luc reporter genes (0.3 μg), β-galactosidase (0.05 μg), and an empty vector (pSuper vector) or increasing amounts of shCypA. The cells were then stimulated with or without BMP4 for 36 h and luciferase activities were measured. Data are expressed as mean ± SEM of at least three experiments. *** *p* < 0.001, ** *p* < 0.01, and * *p* < 0.05 compared to the control group.

To confirm our hypothesis, we designed a *CypA* knockdown plasmid. Knockdown of *CypA* significantly suppressed the expression of endogenous CypA in C2C12 cells (Supplementary Figure S2). Knockdown of *CypA* significantly suppressed BMP4-induced ALP activity examined by ALP staining (Figure 2A,B). Furthermore, knockdown of *CypA* inhibited luciferase activity driven by *ALP*, *BSP*, and *OC* promoters (Figure 2C). These results indicate that the knockdown of *CypA* inhibits osteoblast differentiation.

### 2.3. CypA Interacts with Runx2

**Figure 3:**
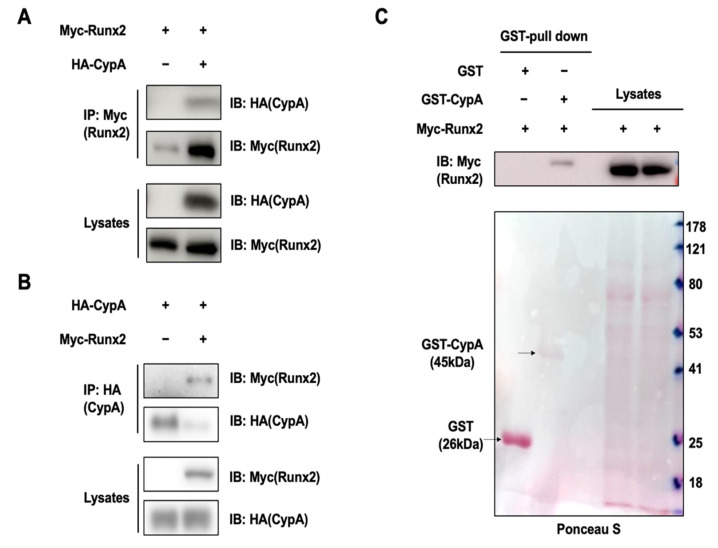
CypA interacts with Runx2. (**A**,**B**) HEK293 cells were transfected with each HA-tagged CypA and/or Myc-tagged Runx2, immunoprecipitation was performed with Myc antibody and immunoblotting with HA or Myc antibody (top panel). The expression of each CypA lysate or Runx2 is identified by the HA antibody or Myc antibody (bottom panel). (**C**) Purified GST-CypA directly interacts with Runx2 (top panel). The purified control (GST-vector) or GST-CypA was identified by ponceau S (bottom panel).

To identify the underlying mechanism, we performed co-immunoprecipitation experiments to test whether CypA can interact with these transcription factors. Myc-Runx2 was expressed alone or together with CypA in HEK293 cells, followed by immunoprecipitation. We observed that CypA interacted with the specific transcription factor Runx2 (Figure 3A,B). We then examined whether CypA can directly bind to Runx2 using the Glutathione S-transferase (GST) pull-down assay. Purified GST-CypA can bind to Runx2 (Figure 3C). These results indicate that the CypA promotes osteoblast differentiation by regulating Runx2.

### 2.4. The Runt and C-terminal Domains of Runx2 Are Essential for Interacting with CypA

**Figure 4:**
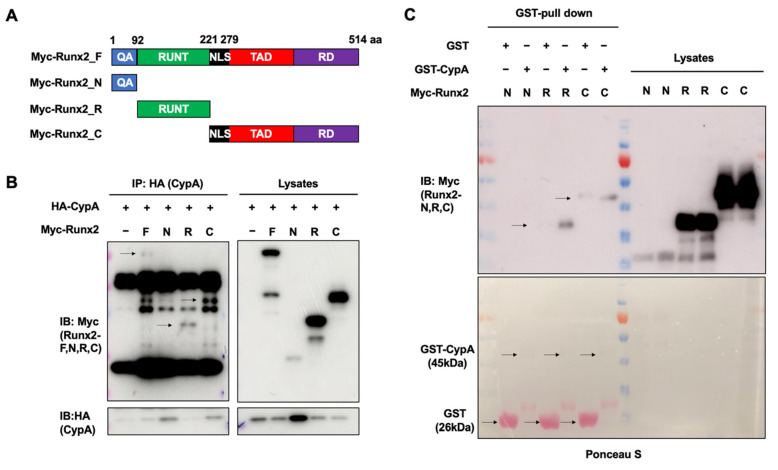
The Runt and C-terminal domains of Runx2 are essential for interacting with CypA. (**A**,**B**) HEK293 cells were transfected with each HA-tagged CypA and/or Myc-tagged Runx2 deletion mutants, immunoprecipitation was performed with HA antibodies, and immunoblotting was performed with HA or Myc antibodies (left panel). The expression of each CypA lysate or Runx2 deletion mutant is identified by HA or Myc antibodies (right panel). (**C**) Purified GST-CypA directly interacts with Runx2 (top panel). The purified control (GST-vector) or GST-CypA was identified by ponceau S (bottom panel).

Runx2 contains a glutamine/alanine-rich domain at its N-terminal that acts as a trans-activating domain, a Runt domain responsible for binding to the transcriptional co-activator core-binding factor-β (CBF-β), and a proline/serine/threonine (PST)-rich domain at its C-terminal end, the PST domain, which functions as a trans-activator and transcriptional repressor [7,11]. By using Runx2 deletion mutants to elucidate the binding site of CypA on Runx2, and immunoprecipitation with CypA separately, we found that the C-terminal and Runt domains of Runx2 can bind CypA (Figure 4A,B). Furthermore, we utilized purified GST-CypA interaction with Runx2 deletion mutants. Purified GST-CypA also binds to the Runt and C-terminal domains of Runx2 (Figure 4C). Taken together, these results suggest that the Runt and C-terminal domains of Runx2 play an important role in the interaction between CypA and Runx2.

### 2.5. CypA Promotes the Osteogenic Activity of Runx2 and Enhances the Binding of Runx2 to Target DNA

**Figure 5:**
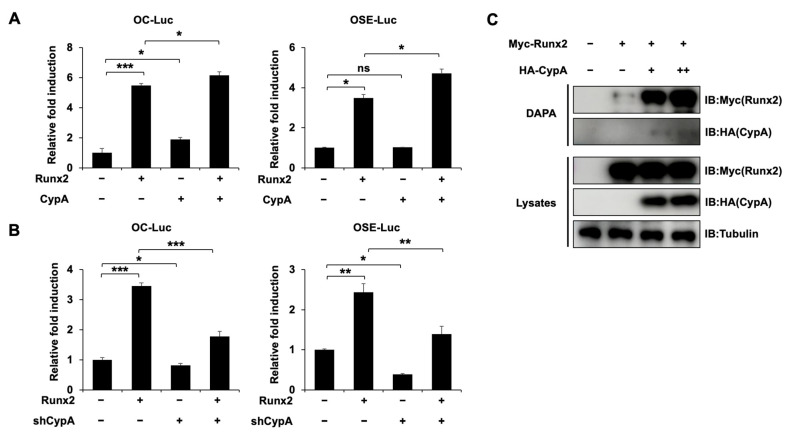
CypA promotes the transcriptional activity of Runx2 by increasing its DNA binding affinity. (**A**,**B**) CypA increases the transcriptional activities of Runx2, while knockdown of *CypA* decreased them. C2C12 cells were transfected with OC-Luc or OSE-Luc reporter gene (0.3 μg), β-galactosidase (0.05 μg) alone or in combination with Runx2 and CypA or shCypA. After 36 h, Luciferase activity was analyzed. Data are expressed as mean ± SEM of at least three experiments. *** *p* < 0.001, ** *p* < 0.01, and * *p* < 0.05 compared to the control group. (**C**) CypA enhances the DNA binding affinity of Runx2. HEK293 cells were transfected with Myc-tagged Runx2 (0.25 μg) and/or increasing amounts of HA-tagged CypA expression plasmid construct. After 48 h, DAPA was performed with the biotinylated OSE2 promoter and Myc or HA antibodies to measure the binding of Runx2 and CypA to the OSE2 promoter region. Tubulin was used as a loading control.

To determine whether CypA affects Runx2 transcriptional activity, the luciferase assay was used. First, Runx2 transfection alone increased the activity of OC and OSE-Luc. After co-transfection with CypA, the stimulatory effect of Runx2 on the OC and OSE promoter activity significantly increased (Figure 5A). Second, the knockdown of *CypA* decreased Runx2-induced OC and OSE luciferase activity (Figure 5B). These results demonstrate that CypA further increases the transcriptional activity of Runx2. It is clear from previous studies that Runx2 plays an important role in osteoblast differentiation through the highly conserved Runt homologous DNA binding domain to OSE2, which is present in osteogenic marker promoters [6,7]. Based on this study, we examined whether CypA can regulate the ability of Runx2 to bind target DNA (OSE2 element). DAPA assay showed that the binding of Runx2 to OSE2 increased as the amount of CypA was increased (Figure 5C). These results suggest that CypA promotes the osteogenic activity of Runx2 through enhancing the DNA binding affinity of Runx2.

### 2.6. Akt Signaling Is Involved in CypA-Regulated Osteoblast Differentiation

**Figure 6:**
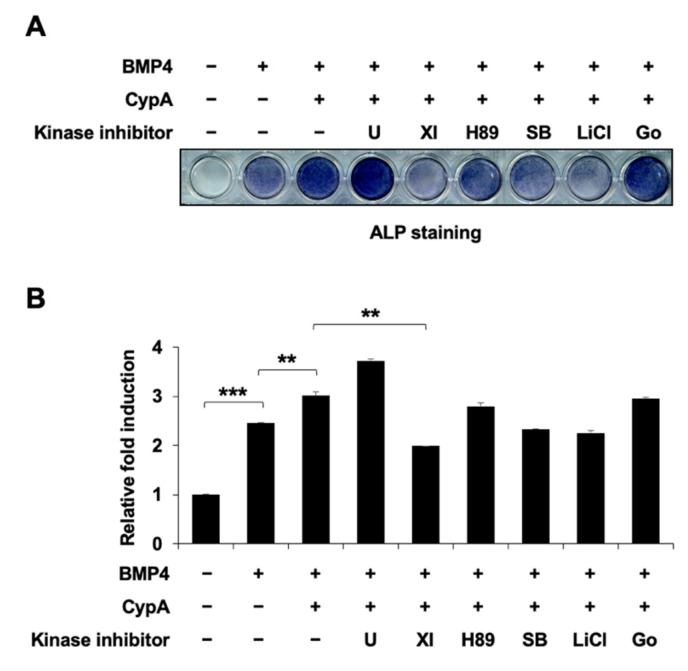
Akt signaling is a major upstream target for CypA function in BMP4-induced osteoblast differentiation. (**A**,**B**) The Akt signaling pathway plays an important role in the CypA-promoted osteoblast differentiation. C2C12 cells were transfected with CypA (0.5 μg) and differentiated in DMEM containing 2% FBS treated with or without BMP4, and treated with the various kinase inhibitors. (**A**) After 72 h of treatment, the ALP staining was performed to detect ALP activity. (**B**) Quantification of ALP staining was performed at an absorbance of 480 nm. Data are expressed as mean ± SEM of at least three experiments. *** *p* < 0.001 and ** *p* < 0.01 compared to the control group. U (U0126; 10 µM; MEK inhibitor), H89 (5 µM; PKA inhibitor), Go (Go6976; 5 µM; PKC inhibitor), SB (SB203580; 5 µM; p38 MAPK inhibitor), XI (5 µM; Akt inhibitor), and LiCl (1 mM; GSK3β inhibitor).

**Figure 7:**
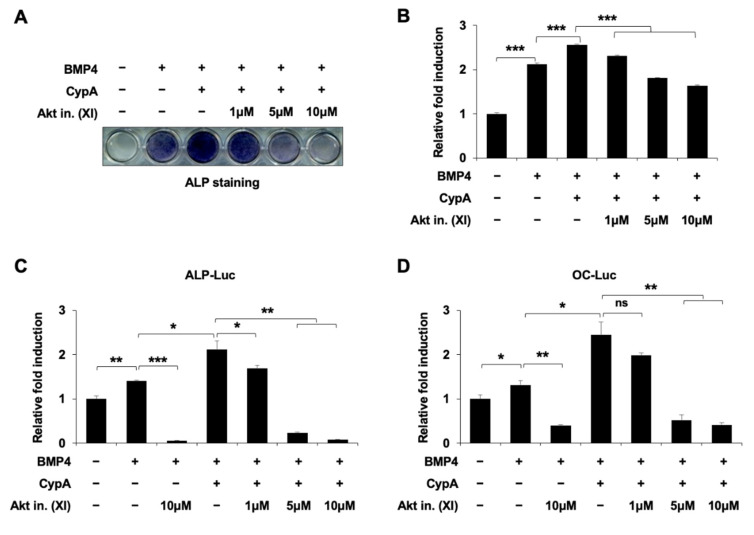
Akt signaling is essential for CypA function in BMP4-induced osteoblast differentiation. (**A**,**B**) An Akt signaling inhibitor dose-dependently decreased CypA-induced ALP activity. C2C12 cells were transfected with CypA (0.5μg) and differentiated in DMEM containing 2% FBS treated with or without BMP4, and treated with Akt inhibitor XI. After 72 h of treatment, the ALP staining was performed to detect ALP activity. (**C**,**D**) Akt signaling regulates CypA-induced promoter activity. C2C12 cells were transfected with ALP-Luc, OC-Luc reporter gene (0.3 μg) alone or in combination with the plasmid construct expressing CypA (0.5 μg). After 24 h of transfection, the cells were changed to differentiation medium (containing 2% FBS) and treated with or without BMP4 and co-treated Akt inhibitor (XI). After 24 h of treatment, luciferase activity was measured. Data are expressed as mean ± SEM of at least three experiments.*** *p* < 0.001, ** *p*< 0.01, and * *p* < 0.05 compared to the control group.

Osteoblast differentiation is regulated by a variety of signaling pathways, including p38 MAPK [12], ERK [13], PKA [14], PKC [15], Akt [16], and GSK3β [17], in addition to the BMP4 signaling pathway [18]. To elucidate the signaling pathways involved in CypA regulation of osteoblast differentiation, we used the ERK pathway-specific inhibitor U0126, the PKA pathway inhibitor H89, the PKC pathway inhibitor Go6976, the p38MAPK pathway inhibitor SB203580, the GSK3β pathway inhibitor LiCl, and the Akt signaling inhibitor XI. Among these kinase inhibitors, we found that the Akt signaling inhibitor XI significantly reduced CypA-stimulated osteoblast differentiation (Figure 6A,B). For examination of the Akt signaling effects on CypA-induced osteoblast differentiation, we treated the Akt signaling inhibitor XI and examined the ALP activity. Akt inhibitors reduced the CypA-induced ALP activity in a dose-dependent manner (Figure 7A,B). In addition, Akt signaling also affected CypA-enhanced osteogenic marker promoter activity (Figure 7C,D). These results suggest that Akt signaling is involved in CypA-stimulated osteoblast differentiation.

## 3. Discussion

Runx2 is one of several transcription factors required for osteoblast differentiation. It activates markers of osteoblast differentiation by binding to the *cis*-acting element OSE in the promoter regions of *ALP*, *collagen*, *BSP*, *OPN*, and *OC* [19,20]. The activity of Runx2 is regulated by post-translational modifications such as acetylation [21], phosphorylation [11], and ubiquitination [22]. Several reports have shown that the ubiquitin/proteasome pathway results in Runx2 degradation, while Akt signaling is involved in Smurf protein-mediated degradation of Runx2. Akt signaling has an essential role in bone remodeling through the regulation of both osteoblasts and osteoclasts [16].

CypA is a member of the PPIase family and a major intracellular target of CsA. The CypA–CsA complex is involved in the activities of the immune system [23]. Several studies have indicated that CypA is expressed both intracellularly and extracellularly and that it plays an important role as a novel molecular timer in tissues such as cartilage and bone [24], thereby influencing cellular processes. Pin1 is another member of the PPIase family that has been extensively studied. Recent evidence suggests that Pin1 has essential roles in skeletal development and osteoblast differentiation by regulating Runx2 [25,26]. The prolyl isomerization effects of Pin1 enhanced the stability and transcriptional activity of Runx2 [25,26]. Although previous research has been carried out on CypA regulating both osteoblasts and osteoclasts, the underlying mechanism is still elusive.

In our study, overexpression of *CypA* exhibited a positive effect on BMP4-induced C2C12 cell differentiation, dramatically enhancing luciferase activity driven by osteogenesis marker genes such as *ALP*, *BSP*, and *OC* promoters (Figure 1). Knockdown of *CypA* reduced BMP4-induced ALP activity and attenuated luciferase activity of *ALP*, *BSP*, and *OC*, indicating that CypA has important roles in osteoblast differentiation (Figure 2). To elucidate the underlying mechanism of how CypA regulates osteoblasts differentiation, we investigated whether CypA interacts with the representative transcription factors (Runx2 and Osterix) involved in osteoblast. Interestingly, we found that CypA specifically interacts with Runx2, but not with Osterix (Figure 3). In the case of Pin1, it positively regulates osteoblast differentiation by interacting with both Runx2 and Osterix [25,26,27]. Furthermore, we found that CypA specifically binds to the Runt domain and C-terminal of Runx2 (Figure 4). Since the Runt domain of Runx2 is responsible for DNA binding [7,11], we assume that the interaction between CypA and Runx2 may affect transcriptional activity by regulating DNA binding affinity. We found that CypA increases the transcriptional activity of Runx2 through enhancing DNA binding affinity (Figure 5). However, it is still not clear whether the PPIase activity of CypA is responsible for Runx2 regulation. Further study is needed for validation on whether CypA inhibitor or PPIase mutant of CypA could abolish CypA function in osteoblast differentiation.

To investigate the upstream signaling involved in CypA-regulated osteoblast differentiation, we used several kinase inhibitors for validation. We found that Akt inhibitor (XI) treatment most effectively suppressed CypA-induced osteoblast differentiation (Figure 6), suggesting that Akt signaling is a major upstream signaling pathway for CypA function in osteoblast differentiation. It has been reported that Akt signaling promotes osteoblast differentiation and increases Runx2 stability and transcriptional activity by enhancing the DNA binding affinity of Runx2 [16,28]. Recent studies have shown that direct phosphorylation of Runx2 by Akt kinase affects the intrinsic DNA binding activity of Runx2, revealing sites that phosphorylate Runx2 in vitro, including the Runt structural domain of Runx2 [29,30,31]. It has also been reported that Runx2 protein has three Akt phosphorylation sites at the Runt domain. The phosphorylation of key residues by Akt signaling is required for the DNA binding activity of Runx2 [29]. Thus, we assume that Akt phosphorylation sites at the Runt domain may be responsible for CypA-Runx2 interaction and CypA regulation.

## 4. Materials and Methods

### 4.1. Plasmids and Chemicals

Plasmids coding HA-tagged CypA and Myc-tagged Runx2 were constructed in a CMV promoter-derived mammalian expression vector (pCS4-3HA,-3Myc). For CypA gene silencing, small hairpin RNA (shRNA) oligonucleotides were synthesized that targeted a 21-base pair (bp) sequence (CCA GCA AGA TCA CCA TTT) of the mouse *CypA* gene. The annealed oligonucleotides were ligated into the pSuper retro puro vectors (#VEC-PRT-0002; Oligoengine, Seattle, WA, USA). All the other experimental reagents purchased from Calbiochem (San Diego, CA, USA) are listed in Table 1.

### 4.2. Cell Culture and Osteoblast Differentiation

Human embryonic kidney cells (HEK293) and C2C12 cells were maintained in Dulbecco’s modified Eagle medium (DMEM) (#12100046; Gibco™, Carlsbad, CA, USA) containing 10% FBS (#S001-07; Welgene Inc., Deagu, Korea) and 1% antibiotic-antimycotics (#15240062; Gibco™) and cultured in an incubator containing 5% CO_2_ at 37 °C, and 80–90% confluent cells were passaged every two days. Stimulation of C2C12 cell differentiation was achieved by culturing the cells until they were fully confluent, discarding the maintenance medium for DMEM containing 2% FBS, and then treating with BMP4.

### 4.3. Alkaline Phosphatase (ALP) Staining

After 72 h of differentiation in 24-well plates, C2C12 cells were washed with phosphate-buffered saline (PBS) and fixed with 4% paraformaldehyde (PF) for 15–30 min at room temperature, followed by the addition of 300 μL of 1-Step™ NBT/BCIP Substrate Solution (#34042; Thermo Scientific, Waltham, MA, USA) for 15 min at room temperature in the dark. The differentiated cells were then stained blue or purple and the alkaline phosphatase staining was quantified by measuring absorbance at 480 nm in triplicate.

### 4.4. Luciferase Assay and Transfection

C2C12 cells and HEK293 cells were transfected using the polyethyleneimine (PEI, #25414; Polysciences, Warrington, UK) transient transfection method, with empty vector-transfected cells used as a control group. Cells were cultured to a density of 50–70% when the plasmids were transfected and changed to fresh medium after 4–6 h. To determine luciferase reporter gene activity, CypA and Runx2 expression constructs, luciferase reporter genes (*ALP*-Luc, *BSP*-Luc, *OC*-Luc, OSE-Luc) 0.3 μg, and β-galactosidase plasmid (internal control) 0.05μg were co-transfected into C2C12 cells. Twenty-four hours after transfection, the cells were treated with BMP4 and the indicated luciferase assay was performed using a luciferase reporter gene test kit (#E1501; Promega, Madison, WI, USA) to measure luciferase activity with a TriStar ^2^ Multimode Reader apparatus. All experiments were performed in triplicate.

### 4.5. Immunoblotting and Immunoprecipitation

Cells were washed with PBS and lysed in ice-cold lysis solution (25 mM HEPES (pH 7.4), 150 mM NaCl, 1% NP-40, 0.25% sodium deoxycholate (Na-Doc), 10% Glycerol, 25 mM NaF, 1 mM EDTA, 1 mM Na_3_VO_4_, 250 μM PMSF, 10 μg/mL leupeptin, 10 μg/mL aprotinin, and 10 μg/mL peptidase) for 15 min, kept on ice for 10 min. The samples were centrifuged at 13,000 rpm at 4 °C and then the supernatant was removed into a new tube. Proteins were quantified according to the Bradford method and then loaded onto sodium dodecyl sulfate-polyacrylamide gels for electrophoresis (SDS-PAGE) and transferred to polyvinylidene difluoride membranes (Immobilon-P; Millipore, Burlington, MA, USA). After blocking, the membranes were incubated with anti-α-tubulin (#sc-8035) antibodies purchased from Santa Cruz Biotechnology (Santa Cruz, CA, USA). Anti-CypA was purchased from Abcam (#ab41684; Cambridge, UK). Anti-Myc (clone 9E10; #11814150001; 1:1000) and anti-HA (clone 12CA5; #11583816001) were purchased from Roche Applied Science (Indianapolis, IN, USA). Membranes were incubated with the antibody in Tween20/TBS. After washing with buffer and incubation with the appropriate horseradish peroxidase-conjugated secondary antibodies, visualization of the antigenic bands was performed using Immobilon Western Chemiluminescent HRP Substrate (#WBKLS0500; Millipore, Burlington, MA, USA) using an Amersham^TM^ ImageQuant^TM^ 800 biomolecular imager (GE Healthcare Life Sciences, Marlborough, MA, USA). Immunoprecipitation was performed by incubating samples with anti-Myc antibodies for 4 h or overnight, followed by incubation with 40 μL of Protein A Sepharose CL-4B (#17096303; GE Healthcare Life Sciences) for 1–3 h. The bound proteins were then released from the Protein A Sepharose by heating to 100 °C and then detected via immunoblotting.

### 4.6. GST Pulldown Assays

For the interaction assays of GST-proteins with CypA, the pGEX-4T2, and pGEX-4T2-CypA plasmids were transformed into the BL21 *E. coli* strain. The bacterial cells were grown at 37 °C in 100 mL Luria-Bertani broth (#244620; BD Difco, Sparks, MD, USA) containing ampicillin (100 μg/mL). At OD600 of ~0.6 A, the bacterial cells were induced using 0.5 mM isopropyl β-D-thiogalactopyranoside (IPTG; #I1401; Duchefa Biochemie, Haarlem, Netherlands) for 1.5 h at 37 °C. The cells were harvested by centrifugation at 3000 rpm for 15 min and resuspended in 5 mL RB buffer (20 mM HEPES (pH 7.4), 120 mM NaCl, 10% Glycerol, 2 mM EDTA) containing 0.5% NP-40 and 1% TritonX-100. The cells were maintained on ice and lysed by sonication. After sonication, the cells were centrifugation at 3000 rpm for 15 min, bacterial pellets were removed, and the supernatant was incubated overnight with glutathione Sepharose ^TM^ 4B (#17-0756-01; GE Healthcare Life Sciences, Marlborough, MA, USA). After two wash processes, the glutathione-Sepharose beads carrying GST-fusion proteins (GST-CypA) were incubated with total cell lysates for 4 h at 4 °C. The bead-protein complex was washed with the RB buffer, eluted in SDS loading buffer, and analyzed by immunoblotting using each antibody.

### 4.7. DNA Affinity Precipitation Assay (DAPA)

DNA–protein complexes were prepared by overnight incubation of cell lysates (500–1000 μg) with 1 μL of biotinylated DNA probe in the upstream region of −2333 to −2345 of the OSE2 gene translation start site and then precipitated using 50 μL of streptavidin-coated beads (GE Healthcare Life Sciences), as described previously [32]. The DNA–protein complex was washed twice with 1× annealing buffer and twice with 1× gel shift buffer then the bound proteins were washed with PBS and separated by 12% SDS-PAGE, followed by immunoblotting analysis with specific antibodies against HA and Myc. The biotinylated reverse OSE2 sequences were 5′-biotin-GCA AGG CCA CGT GGA GGA CAC GGG-3′ and 5′-biotin-CCC GTG TCC TCC ACG TGG CCT TGC-3′.

### 4.8. Statistical Analysis

All experiments were performed three times with different samples each time, yielding essentially identical results. The standard error of the mean was used to express the results. An independent *t*-test was used to make comparisons between two groups, otherwise one-way analysis of variance (ANOVA) followed by Tukey’s multiple comparison test was used. A probability value of *p* < 0.05 was considered statistically significant.

## 5. Conclusions

Our molecular findings collectively provide a new mechanism for CypA-mediated regulation of osteoblast differentiation, a new factor for Runx2 protein-protein interactions via the Runt domain, and provide evidence for the involvement of an Akt signaling pathway besides BMP signaling in the CypA regulatory process.

## Figures and Tables

**Table 1:** Kinase inhibitor information.

Inhibitor	Product Name	Catalog #
Mitogen-activated protein kinase kinase (MEK) inhibitor	U0126	662005
Protein kinase A (PKA) inhibitor	H89	371963
Protein kinase C (PKC) inhibitor	Go6976	365250
p38 MAPK inhibitor	SB203580	559386
Akt inhibitor	XI	124028
Glycogen synthase kinase 3 (GSK3) inhibitor	LiCl	438002

## Data Availability

Please contact the corresponding author for reasonable data requests.

## Supplementary Materials

The following supporting information can be downloaded at: https://www.mdpi.com/article/10.3390/ijms23169244/s1.
