# Supplementary material for: Cyclophilin A Promotes Osteoblast Differentiation by Regulating Runx2
Source: Int J Mol Sci. 2022 Aug 17;23(16):9244. doi: 10.3390/ijms23169244 (PMC9409320; doi:10.3390/ijms23169244)
Supplement: Supplementary file 1 [file ijms-23-09244-s001.zip › ijms-1858858-supplementary.pdf]

Supplementary materials

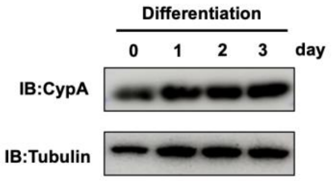

Supprelemantary Figure S1. CypA is ubiquitously expressed during osteoblasts differentiation. C2C12 cells were maintained differentiation medium (containing 2% FBS) treated with BMP4 for 72h. Cell lysates were harvested indicated time.

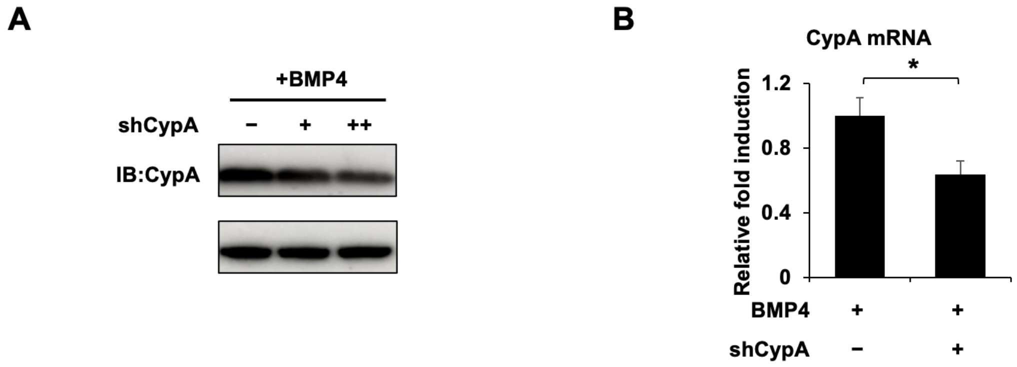

Supprelemantary Figure S2. Knockdown of CypA significantly suppressed the endogenous CypA expression. C2C12 cells were cultured in DMEM (containing 10% FBS) medium and transfected with empty vector or increasing amounts of shCypA plasmid using PEI transfection reagent. After 24h, cells were removed 10% FBS containing DMEM and maintained differentiation medium (including 2% FBS) treated with BMP4 for 48h.
